# Multimodal data-driven prognostic model for predicting long-term outcomes in older adult patients with sarcopenia: a retrospective cohort study

**DOI:** 10.3389/fpubh.2025.1614374

**Published:** 2025-08-07

**Authors:** Mengdie Liu, Wen Guo, Jin Peng, Jinhui Wu

**Affiliations:** Center of Gerontology and Geriatrics, National Clinical Research Center for Geriatrics, West China Hospital, Sichuan University, Chengdu, China

**Keywords:** sarcopenia, machine learning, multimodal data, mortality prediction, nomogram

## Abstract

**Background:**

Sarcopenia (SP) is a progressive, age-related disease that may result in various adverse health outcomes and even mortality in older adults. Accurately predicting the mortality risk of older adults with SP is essential for informed clinical decision-making. This study aims to utilize machine learning techniques that incorporate sociodemographic factors, health-related metrics, lifestyle variables, and biomarker data to improve risk stratification and management in older adults with SP.

**Methods:**

We analyzed data from the NHANES from 1999–2006 and 2010–2018, including a total of 1,619 older adult patients with SP, with a 10-year follow-up period for this population, during which 541 (33%) patients died and 1,078 (67%) survived. This study extracted 36 clinical variables for each patient, encompassing sociodemographic factors, health-related metrics, and biochemical markers. Feature selection was performed using Lasso Regression, XGBoost, and Random Forest machine learning algorithms, and a nomogram model was developed using univariate and multivariate Cox regression analyses, with validation of its accuracy, concordance, and clinical applicability.

**Results:**

A total of 12 feature variables were identified through the combined use of three machine learning methods. Univariate and multivariate Cox regression analyses identified Age, Height, Neutrophil count (NENO), The ratio of hemoglobin to red cell distribution width (HRR), Uric Acid (UA), and Creatinine as significant predictors of mortality in older adults with SP, and a nomogram model was constructed based on these feature variables, with model performance assessed through discrimination, calibration curves, and clinical utility evaluation. The model achieved AUC values of 0.753, 0.773, 0.782, and 0.800 at 1, 3, 5, and 10 years, respectively, demonstrating good concordance and adequate calibration. Decision curve analysis (DCA) indicated that the model had broad applicability in predicting short-term and long-term outcomes in older adult patients with SP. Finally, based on the nomogram risk score, patients were stratified into risk groups and survival curves were plotted, illustrating a significantly lower survival probability in the high-risk group compared to the low-risk group (*p* < 0.0001).

**Conclusion:**

Utilizing advanced statistical and machine learning techniques, we developed and validated a prognostic model for SP in the older adult that integrates multimodal data, enhancing predictive accuracy and reliability. This model provides valuable insights for clinicians, facilitates risk stratification, and provides personalized interventions for older adults with SP.

## 1 Introduction

The human aging process is characterized by alterations in body composition, notably the loss of skeletal muscle mass. Beginning at approximately age 25, both the size and quantity of muscle fibers progressively decline. To define this age-related loss of muscle mass, Rosenberg et al. first introduced the term “sarcopenia (SP)” in 1989 (1). As research progressed, the International Working Group on SP (IWGS) (2) refined and expanded the definition of SP.

SP is a disease characterized by a reduction in whole-body skeletal muscle mass (including skeletal muscle and cardiac muscle) and decreased muscle strength, with or without physical function decline (3). Epidemiological studies indicate that the global prevalence of SP among individuals aged 60 years and older ranges from approximately 10 to 27%, while in those aged 80 years and above, it can increase to nearly 50% (4). A survey of community-dwelling older adults reported a SP prevalence of approximately 10–40% (5, 6). Furthermore, as population aging accelerates, the prevalence of SP continues to rise. By 2050, the population aged 65 and older will reach 2.1 billion, making SP a significant health concern in rapidly aging societies (7). Additionally, SP is associated with an increased risk of adverse events, including falls, fractures, frailty, mobility impairment, disability, complications, infections, metabolic disorders, reduced quality of life, and mortality (3, 8–10). These consequences impose a substantial burden on healthcare resources and society at large. For example, Janssen et al. reported that SP contributes to an additional annual healthcare expenditure of $18.4 billion in the United States ($10.8 billion for men and $7.7 billion for women), representing approximately 1.5% of total healthcare costs (11). These findings underscore SP as a critical public health concern among older adults, highlighting the pressing challenge of its prevention and mitigation.

Accurate mortality risk prediction in older adult SP patients is crucial for effective therapeutic decision-making and risk management. Although several SP risk prediction models exist, they all have certain limitations (12). For instance, some models rely on difficult-to-collect and time-consuming predictors, limiting their utility in clinical practice. Other models fail to capture all risk factors for SP, potentially compromising their predictive accuracy. These gaps underscore the necessity for integrated multidimensional prediction systems to guide personalized therapeutic approaches and improve clinical outcomes in geriatric SP.

The progression of SP is influenced by various etiological variables, including systemic inflammation, neuromuscular function loss, decreased anabolic hormone levels, mitochondrial dysfunction, and oxidative stress (13–15). These factors affect skeletal muscle structure and function in older adults, leading to SP development, progression, and even mortality. Emerging consensus indicates that age-related chronic subclinical inflammation is a pivotal driver in SP pathophysiology (16). Nevertheless, current research predominantly emphasizes canonical inflammatory biomarkers (17–20)(IL-6, TNF-α, CRP) while overlooking the prognostic significance of alternative cytokine networks. Therefore, this study will incorporate other specific biomarkers to investigate their association with all-cause mortality and establish a more comprehensive and accurate prognostic model.

This study utilizes data from an 8-year cohort study to investigate the relationships between biomarkers, risk factors, and SP mortality. It specifically aims to identify key biomarkers linked to SP mortality. It constructs a long-term prognostic model by integrating multimodal data with multiple Machine Learning (ML) approaches to predict the mortality risk in older adult patients with SP. Furthermore, we assess the effectiveness of the model in individualized risk stratification for SP patients based on the identified factors, ultimately aiming to improve patient prognosis.

## 2 Methods

### 2.1 Data source

This study utilizes data from the National Health and Nutrition Examination Survey (NHANES), a nationally representative program in the United States. The survey adopts a multistage, stratified probability sampling design incorporating cluster sampling to represent the population’s characteristics comprehensively. Data were collected using standardized interviews, physical examinations, and laboratory testing, systematically capturing multidimensional health data, including demographic characteristics, dietary intake, medical history, laboratory test results, and survey responses.^1^ The research protocol received formal approval from the National Center for Health Statistics Ethics Review Board. Written informed consent was obtained from all enrolled participants prior to their involvement in the study.

### 2.2 Study population

This study analyzed NHANES population data from eight cycles spanning 1999–2018, explicitly encompassing data from 1999–2000, 2001–2002, 2003–2004, 2005–2006, 2011–2012, 2013–2014, 2015–2016, and 2017–2018 cycles. The investigation specifically targeted individuals aged ≥60 years who possessed comprehensive datasets encompassing appendicular skeletal muscle mass (ASM) measurements, anthropometric parameters, laboratory-derived biomarkers, and longitudinal survival tracking records.

### 2.3 Outcome events

This research defined all-cause mortality as the principal endpoint in older adults diagnosed with SP. Survival status verification was conducted via linkage to the National Death Index (NDI). The follow-up period was accurately measured as the time (in months) from the baseline survey date to either the date of death or the end of follow-up (December 31, 2019).

### 2.4 Data structure

1. Inclusion Criteria: Age ≥60 years; Complete DXA scan data, including ASM, height and weight; Complete biomarker data related to inflammation.; Valid survival follow-up records; Muscle mass, muscle strength, and muscle strength per unit size constitute essential diagnostic components of sarcopenia. Grip strength measurement utilizes thresholds of <28.0 kg (men) and <18.0 kg (women) to identify low muscular strength, a key presarcopenia criterion. Based on the guidelines from the Foundation for the National Institutes of Health (FNIH), individuals in the NHANES database were classified as having SP if their BMI-adjusted skeletal muscle mass index (ASM/BMI) was <0.512 for females or <0.789 for males (21).
2. Exclusion Criteria: Individuals with incomplete DXA data; Pregnant individuals; Participants with incomplete relevant laboratory data.

**Figure 1:**
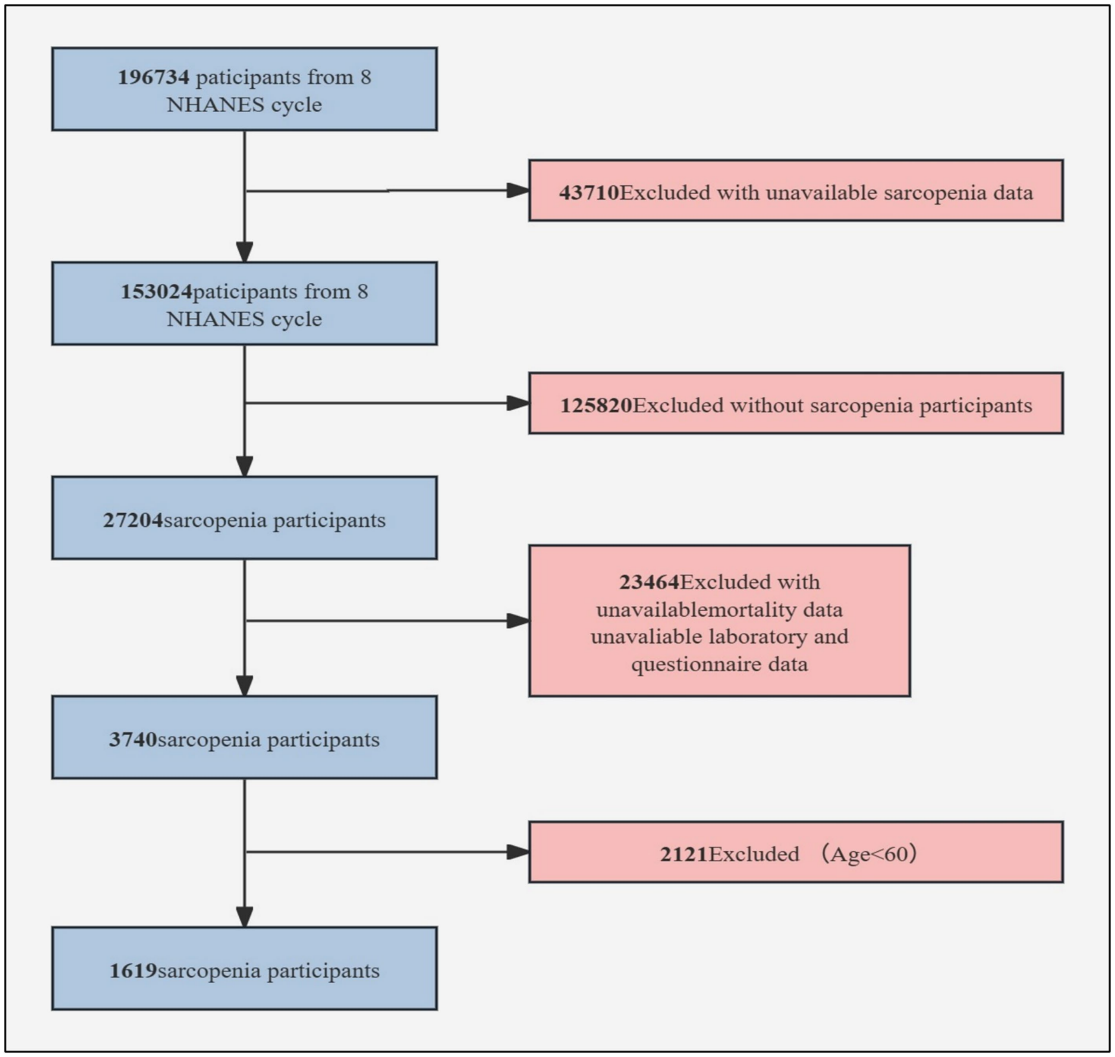
Sample selection flowchart for NHANES 1999–2018.

As shown in Figure 1, after screening, our final study cohort included 1,619 SP patients with complete survival analysis data.

### 2.5 Data information

We employed a longitudinal cohort design, collecting baseline participant biomarker data and following them over time to evaluate the risk of SP-related mortality. Multiple variables were integrated into the predictive model to enhance its accuracy. The variables considered included: (1) Demographic characteristics: Age, Gender, Race, Marital status, and income level (PIR). (2) Anthropometric measurements: Height, Weight, Waist Circumference(WC), and Body Mass Index (BMI). (3) Health behaviours: Smoking, Obesity, diabetes, and Hypertension. (4) Laboratory indices: Blood samples from all participants were collected at the NHANES Mobile Examination Center (MEC).

The MEC used the Coulter HMX (Coulter Electronics Ltd., Bedfordshire, UK) and Beckman Coulter DXH 800 (Beckman Coulter, Brea, CA, USA) for complete blood count analysis, including neutrophil count (NENO), lymphocyte count (LYMNO), platelet count (PLT), hemoglobin (HGB), and red cell distribution width (RDW). The DcX800 chemistry analyzer was employed to assess biochemical markers, including albumin, alanine aminotransferase (ALT), aspartate aminotransferase (AST), and uric acid (UA). The Cobas 6,000 Chemistry Analyzer was utilized to quantify total cholesterol (TC), low-density lipoprotein cholesterol (LDL-c), and high-density lipoprotein cholesterol (HDL-c).

Serum-related biomarkers were calculated based on laboratory parameters measured at the MEC: NLR (22) (Neutrophil to lymphocyte ratio), PLR (23) (Platelet to lymphocyte ratio)、HRR (The ratio of hemoglobin to red cell distribution width), SII (24) (Systemic immune inflammation index), CRI-I (25) (Castelli risk index I), CRI-II (25) (Castelli risk index II), NHHR (26) (non-high-density lipoprotein cholesterol to high-density lipoprotein cholesterol ratio), UHR (27) (uric acid to high-density cholesterol ratio), ALI(Advanced lung cancer inflammation index) and BRI (28) (Body roundness index).

NLR = Neutrophil count (cell/ml) / Lymphocyte count (cell/ml).

PLR = Platelet count (cell/ml) / Lymphocyte count (cell/ml).

HRR = Hemoglobin (g/dl) / Red cell distribution width (%).

SII=Platelet count (cell/ml) × Neutrophil count (cell/ml) / Lymphocyte count (cell/ml).

CRI-I = Total cholesterol (mmol/L) / High density lipoprotein cholesterol (mmol/L).

CRI-II = LDL-c (mmol/L) / HDL-c (mmol/L).

NHHR = [TC (mmol/L)-HDL-c (mmol/L)] / HDL-c(mmol/L).

UHR = UA (umol/L) / HDL-c (mmol/L).

ALI = [Albumin (g/dl) × BMI (kg/m2)] / NLR (cell/ml).

BRI = 364.2–365.5 $\sqrt{1-(\frac{(\mathrm{WC}(m)/{\left(2\pi \right)}^{2}}{{\left(0.5\times \text{Height}(m)\right)}^{2}})).}$

### 2.6 Variable screening

In this study, we employed three different machine learning algorithms—LASSO regression, XGBoost and Random Forest- to select features. First, we implemented LASSO regression, which applies an L1 regularization constraint to shrink regression coefficients of low-contribution features to zero, achieving effective dimensionality reduction while controlling the risk of overfitting. Next, we utilized the XGBoost algorithm to evaluate feature importance. By optimizing the distributed gradient boosting algorithm and incorporating regularization terms and custom loss functions, the generalization ability of the model was significantly enhanced. Finally, we applied the RF ensemble algorithm for survival analysis modelling and feature importance assessment. We internally validated the model’s generalization ability by generating decision trees and calculating the Out-of-Bag (OOB) error, thereby identifying the most critical features. The standard features identified by all three algorithms resulted in 12 prognostic markers for predicting SP patient survival. Additionally, univariate and multivariate Cox regression analyses were performed to verify whether the selected features were independent prognostic factors in SP patients beyond other clinical variables. Ultimately, Age, Height, NENO, UA, Creatinine and HRR were confirmed as independent prognostic factors for mortality risk in SP patients.

### 2.7 Assessment of mortality

The performance of the nomogram model was assessed using three approaches: discrimination, calibration curve, and clinical utility. The model’s discriminative ability was analyzed using the area under the ROC curve. Furthermore, Bootstrap resampling was performed to refine the C-index, providing an estimate of the model’s predictive accuracy in future scenarios. Calibration curve analysis was subsequently conducted to evaluate the predictive model’s accuracy alignment between observed outcomes and probabilistic estimates. Finally, decision curve analysis (DCA) was performed to examine the net benefit of the model across various threshold probabilities. The nomogram risk score was calculated for each patient, and patients were classified into low-risk and high-risk groups based on median survival. Kaplan–Meier analysis was employed to compare survival distributions between the high-risk and low-risk groups.

### 2.8 Statistical analysis

In addressing potential estimation distortions induced by partial data availability, covariates demonstrating missingness rates surpassing 20% were methodologically omitted at the preliminary stage of information acquisition. An advanced multiple imputation framework was then operationalized to manage remaining data gaps within preserved parameters, following a rigorously structured implementation sequence: first, comprehensive specification of interdimensional associations using iterative conditional modeling; second, probabilistic assignment of multiple coherent replacement values through regression-based pattern alignment; third, generation of five computationally compatible synthetic datasets. This Bayesian-consistent framework maintains stochastic characteristics while incorporating measurement variability following Rubin’s variance estimation principles, ultimately optimizing inferential precision and analytical robustness in subsequent quantitative investigations.

Statistical analysis of the study results was performed using R software 4.2.2. The following R packages were employed: “tidyverse” for data manipulation, “glmnet” for Lasso regression-based feature selection, and “survival” for survival modeling. “xgboost” for variable selection via Extreme Gradient Boosting; “randomForestSRC” for random survival forest analysis with embedded feature selection; “ggplot2,” “ggvenn,” “ggrain,” and “ggDCA” for data visualization; “timeROC” and “survivalROC” for generating time-dependent ROC curves. Initially, the Shapiro–Wilk test was conducted to assess the normality of the data distribution. Continuous variables were presented as means±SD or median with 25th and 75th Percentiles. Group comparisons were analyzed using Student’s t-test (normally distributed continuous variables) or Mann–Whitney U test (non-normally distributed data). Categorical variables were reported as frequency counts with percentages, and intergroup differences were assessed via the Chi-square test or Fisher’s exact test. A two-tailed significance threshold of *p* < 0.05 was applied for all statistical inferences.

## 3 Results

### 3.1 Analysis of baseline data

After screening, the final cohort included 1,619 older adult patients with SP, among whom 541 (33%) died and 1,078 (67.0%) survived. Table **1** presents the baseline characteristics of the deceased and surviving SP patients. And present the distribution characteristics of the variable in the Alive group and the Dead group using box plots (Supplementary Figure 1a). Univariate analysis revealed elevated mortality risk in sarcopenia patients with: Age (66y vs. 75y, *p* < 0.001), female sex (54% vs. 42%, *p* < 0.001), Mexican ethnicity (50% vs. 28%, *p* < 0.001), married status (69% vs. 55%, *p* < 0.001), higher BMI (30.5 vs. 28.8), obesity (54% vs. 40%, *p* < 0.001), elevated ALT (22 U/L vs. 19 U/L, *p* < 0.001), increased platelet count (261 × 10^9^/L vs. 241 × 10^9^/L), lymphocyte count (2.0 vs. 1.8 × 10^9^/L), and higher ALI levels. Paradoxically younger age in decedents (66 year vs. 75 year, *p* < 0.001) reflects the shifting epidemiology toward younger-onset sarcopenia, implying multifactorial mortality mechanisms beyond chronological age (29). Female sarcopenia patients demonstrate worse outcomes, potentially mediated by hormonal dynamics (30). Sarcopenic obesity confers elevated mortality risk, consistent with JAMA evidence linking it to reduced survival (31). PLR, ALT, and ALI levels reflect systemic inflammation. ALI integrates inflammatory and nutritional status, capturing complex interplay between systemic inflammation, immune function, and nutritional state. ALI has emerged as a promising prognostic marker in cancer (32), CVD, and chronic inflammatory disorders (33). Thus, inflammation may critically influence sarcopenia prognosis in our cohort. Unexpectedly lower creatinine in decedents (70.7 vs. 79.6 μmol/L, *p* < 0.001) may reflect higher muscle mass in younger subgroups or unmeasured confounders. Furthermore, we performed stratified analyses by sex for key variables, created sex-stratified box plots (Supplementary Figure 1b) to illustrate differences in survival status between genders, and incorporated the results of statistical tests for group comparisons in each plot (as shown in the figure below). In these figures, the impacts of age, height, BMI, albumin, ALT, etc., on survival outcomes may vary between genders (*p* < 0.05).

**Table 1:** Baseline patient characteristics.

Variable	Overall (*N* = 1,619)	Dead (*N* = 541)	Alive (*N* = 1,078)	*p*-value
Age (year)				**<0.001** ^***^
Median (Q1, Q3)	71.0 (65.0, 80.0)	66.0 (62.0, 70.0)	75.0 (69.0, 82.0)	
Gender, *n* (%)				**<0.001** ^***^
Male	874 (54%)	247 (46%)	627 (58%)	
Female	745 (46%)	294 (54%)	451 (42%)	
Race, *n* (%)				**<0.001** ^***^
Mexican American	576 (36%)	270 (50%)	306 (28%)	
Non-Hispanic White	841 (52%)	201 (37%)	640 (59%)	
Non-Hispanic Black	94 (5.8%)	30 (5.5%)	64 (5.9%)	
Other	108 (6.7%)	40 (7.4%)	68 (6.3%)	
Marital, *n* (%)				**<0.001** ^***^
Median (Q1, Q3)	968 (60%)	372 (69%)	596 (55%)	
Pir income level, *n* (%)				0.11
Poor	291 (18%)	109 (20%)	182 (17%)	
Not poor	1,328 (82%)	432 (80%)	896 (83%)	
Smoke, *n* (%)	60 (3.7%)	14 (2.6%)	46 (4.3%)	0.10
Height (cm)				
Median (Q1, Q3)	160.2 (153.0, 167.0)	158.5 (152.4, 165.4)	161.1 (153.3, 167.6)	**<0.001** ^***^
Body Mass Index (kg/m^2^)				**<0.001** ^***^
Median (Q1, Q3)	29.3 (26.3, 33.4)	30.5 (27.4, 34.4)	28.8 (25.8, 32.5)	
Waist Circumference (cm)				0.3
Median (Q1, Q3)	103.0 (95.3, 112.9)	103.6 (96.0, 112.7)	102.8 (95.0, 112.9)	
Obesity, *n* (%)	724 (45%)	291 (54%)	433 (40%)	**<0.001** ^***^
Diabetes, *n* (%)	535 (33%)	159 (29%)	376 (35%)	0.029
Hypertension, *n* (%)	1,177 (73%)	364 (67%)	813 (75%)	**<0.001** ^***^
Total Cholesterol (mmol/L)				0.5
Median (Q1, Q3)	5.3 (4.6, 6.0)	5.36 (4.6, 6.0)	5.34 (4.6, 6.0)	
High-density lipoprotein cholesterol (mmol/L)				0.2
Median (Q1, Q3)	1.3 (1.1, 1.6)	1.3 (1.0, 1.5)	1.3 (1.1, 1.6)	
Low-density lipoprotein cholesterol (mmol/L)				0.3
Median (Q1, Q3)	3.1 (2.5, 3.8)	3.1 (2.6, 3.8)	3.1 (2.5, 3.8)	
Albumin (g/L)				0.016^*^
Median (Q1, Q3)	42.0 (40.0, 44.0)	42.0 (40.0, 44.0)	42.0 (40.0, 44.0)	
Alanine aminotransferase (U/L)				**<0.001** ^***^
Median (Q1, Q3)	20.0 (16.0, 25.0)	22.0 (18.0, 28.0)	19.0 (15.0, 24.0)	
Aspartate aminotransferase (U/L)				0.5
Median (Q1, Q3)	23.0 (20.0, 27.0)	23.0 (20.0, 27.0)	23.0 (19.0, 27.0)	
Creatinine (umol/L)				**<0.001** ^***^
Median (Q1, Q3)	79.6 (61.9, 97.2)	70.7 (61.9, 88.4)	79.6 (70.7, 97.2)	
Serum Fertin (nmol/L)				0.3
Median (Q1, Q3)	14.3 (10.8, 18.1)	14.5 (11.1, 18.1)	14.1 (10.7, 18.1)	
Lymphocyte Count (10^9^/L)				**<0.001** ^***^
Median (Q1, Q3)	1.9 (1.5, 2.4)	2.0 (1.6, 2.5)	1.8 (1.4, 2.3)	
Neutrophil Count (10^9^/L)				0.009^**^
Median (Q1, Q3)	4.3 (3.4, 5.3)	4.2 (3.3, 5.2)	4.4 (3.5, 5.4)	
Hemoglobin (g/dl)				0.004^**^
Median (Q1, Q3)	14.3 (13.3, 15.2)	14.5 (13.6, 15.2)	14.2 (13.2, 15.2)	
Red cell distribution width (%)				**<0.001** ^***^
Median (Q1, Q3)	12.9 (12.4, 13.5)	12.6 (12.2, 13.1)	13.0 (12.4, 13.7)	
Platelet Count (10^9^/L)				<0.001***
Median (Q1, Q3)	249.0 (206.0, 295.0)	261.0 (221.0, 301.0)	241.0 (201.0, 288.0)	
Uric Acid (umol/L)				**<0.001** ^***^
Median (Q1, Q3)	333.1 (285.5, 398.5)	327.1 (273.6, 374.7)	339.0 (285.5, 410.4)	
The ratio of hemoglobin to red cell distribution width				**<0.001** ^***^
Median (Q1, Q3)	1.1 (1.0, 1.2)	1.1 (1.1, 1.2)	1.1 (1.0, 1.2)	
Neutrophil to lymphocyte ratio				**<0.001** ^***^
Median (Q1, Q3)	2.3 (1.7, 3.1)	2.0 (1.6, 2.7)	2.4 (1.8, 3.2)	
Platelet Lymphocyte Ratio				0.12
Median (Q1, Q3)	131.3 (100.8, 171.3)	128.5 (101.5, 161.3)	132.8 (100.0, 175.3)	

Systemic immune inflammation index				0.018^*^
Median (Q1, Q3)	550.8 (390.8, 788.9)	537.3 (384.8, 734.7)	559.5 (394.3, 829.3)	
Castelli risk index I				0.079^*^
Median (Q1, Q3)	4.0 (3.3, 5.0)	4.2 (3.4, 5.1)	4.0 (3.2, 5.0)	
Castelli risk index II				0.090^*^
Median (Q1, Q3)	2.4 (1.8, 3.2)	2.5 (1.8, 3.2)	2.3 (1.7, 3.1)	
NHHR				0.079^*^
Median (Q1, Q3)	3.0 (2.3, 4.0)*	3.2 (2.4, 4.1)e	3.0 (2.2, 4.0)*	
Uric acid to high-density cholesterol ratio				0.039^*^
Median (Q1, Q3)	261.5 (194.4, 345.0)	252.9 (196.3, 328.3)	268.9 (194.2, 355.0)	

Advanced lung cancer inflammation index				**<0.001** ^***^
Median (Q1, Q3)	55.1 (39.8, 76.6)	64.1 (47.8, 85.2)	50.6 (36.1, 72.0)	
Body roundness index				0.002^**^
Median (Q1, Q3)	6.5 (5.4, 7.9)	6.6 (5.6, 8.1)	6.4 (5.3, 7.8)	
^1^Wilcoxon rank sum test; Fisher’s exact test				

*p < 0.05, **p < 0.01, ***p < 0.001. Bold value indicates significant difference between the two groups.

Univariate analysis may be susceptible to multicollinearity effects. Consequently, we incorporated the variables into Lasso regression, XGBoost, and random forest algorithms for further feature selection.

### 3.2 LASSO regression feature selection

**Figure 2:**
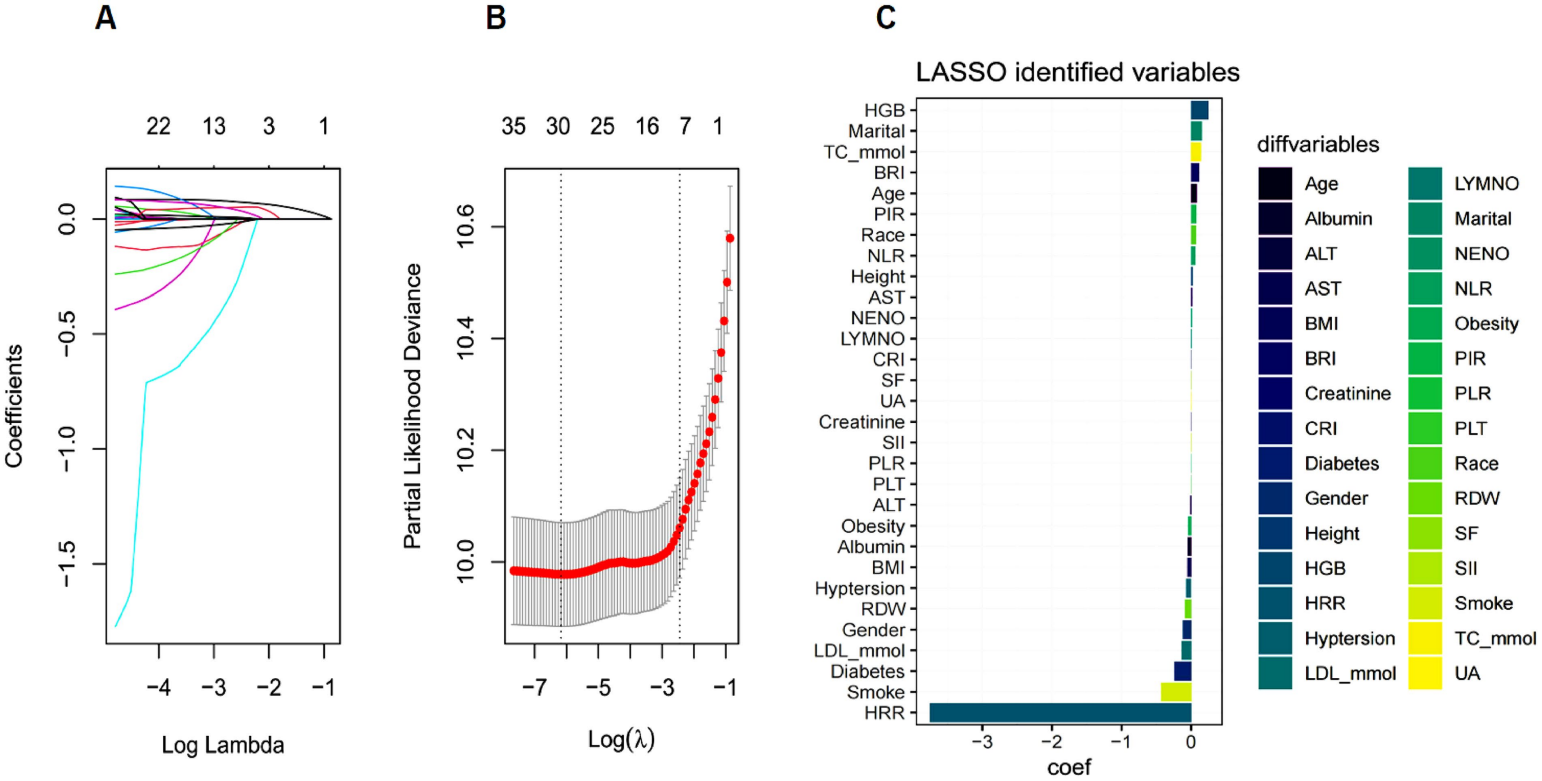
**(A)** The coefficient path diagram of Lasso regression. **(B)** Regularization path analysis diagram. **(C)** Lasso Coefficients Bar Plot.

The “glmnet” package was utilized to model the survival data of SP patients, and 10-fold cross-validation was performed to identify the optimal regularization parameter *λ*. First, the partial likelihood deviance corresponding to different λ values was calculated (Figure 2A). The λ value that resulted in the minimum deviance within one standard error was then selected as the final parameter (Figure 2B). This procedure selected 30 nonzero coefficient variables with significant predictive value, encompassing demographic characteristics (Age, Gender, Race, Marital status), health behaviours (Smoking, Height, BMI, Obesity, Diabetes, Hypertension) and laboratory parameters (TC_mmol, LDL_mmol, Albumin, ALT, AST, Creatinine, SF, LYMNO, NENO, HGB, RDW, PLT, UA, HRR, NLR, PLR, SII, CRI, BRI) (Figure 2C).

### 3.3 XGBoost feature selection

**Figure 3:**
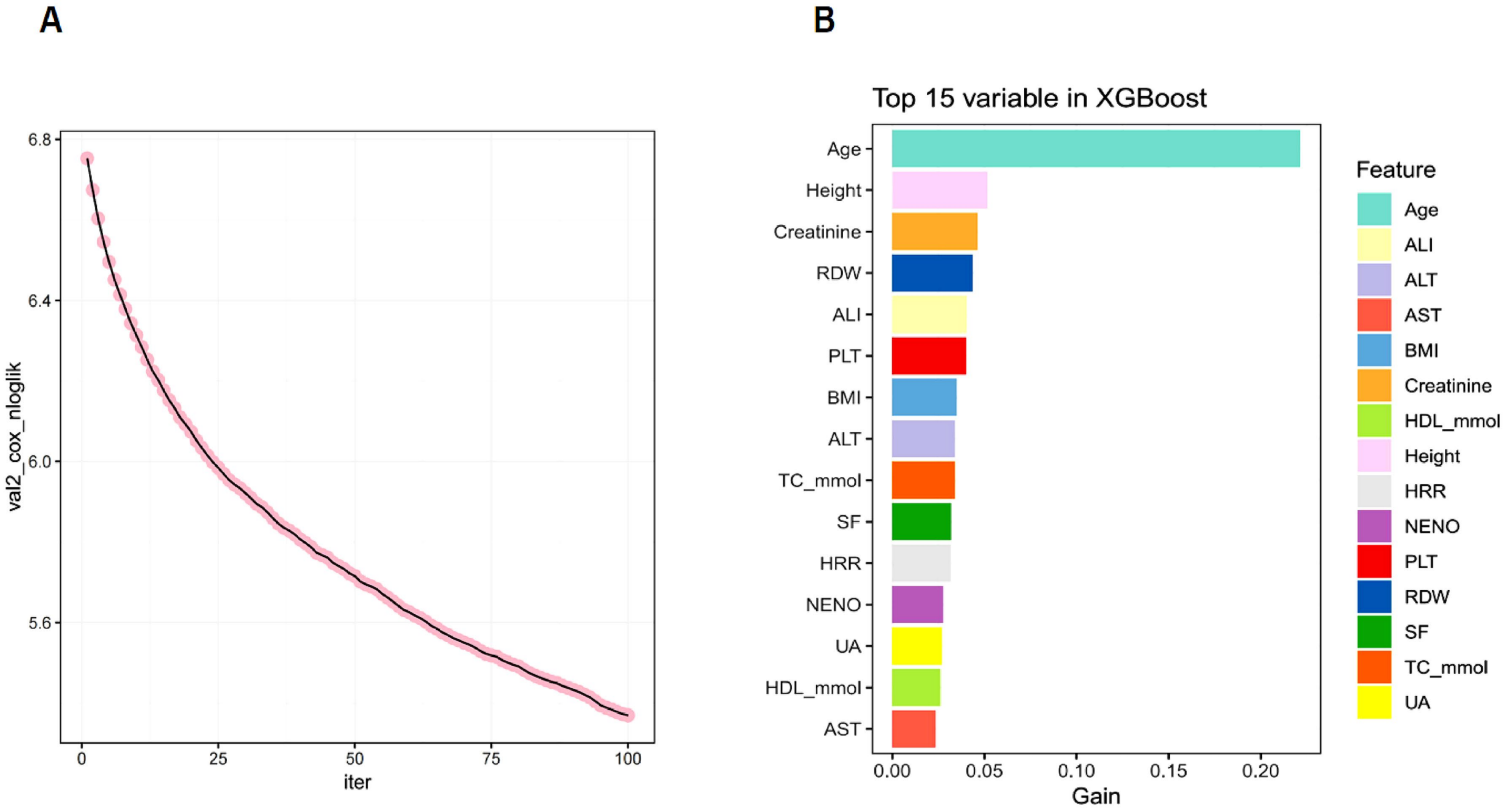
**(A)** XGBoost Training Loss vs. Iteration Curve. **(B)** XGBoost Feature Importance Ranking (Top 15 Variables).

The extreme gradient boosting XGBoost algorithm was used to assess the importance of the feature. Figure 3A. illustrates the dynamic change curve of Cox negative log-likelihood values across iterations (0–100 epochs) during training. At 100 iterations, the validation loss stabilized, suggesting that the model had converged. Figure 3B. displays the top 15 most important features, highlighting Age, ALI, ALT, AST, BMI, Creatinine, HDL_mmol, Height, HRR, NENO, PLT, RDW, SF, TC_mmol and UA as key predictors of SP patient prognosis.

### 3.4 Random Forest feature screening

**Figure 4:**
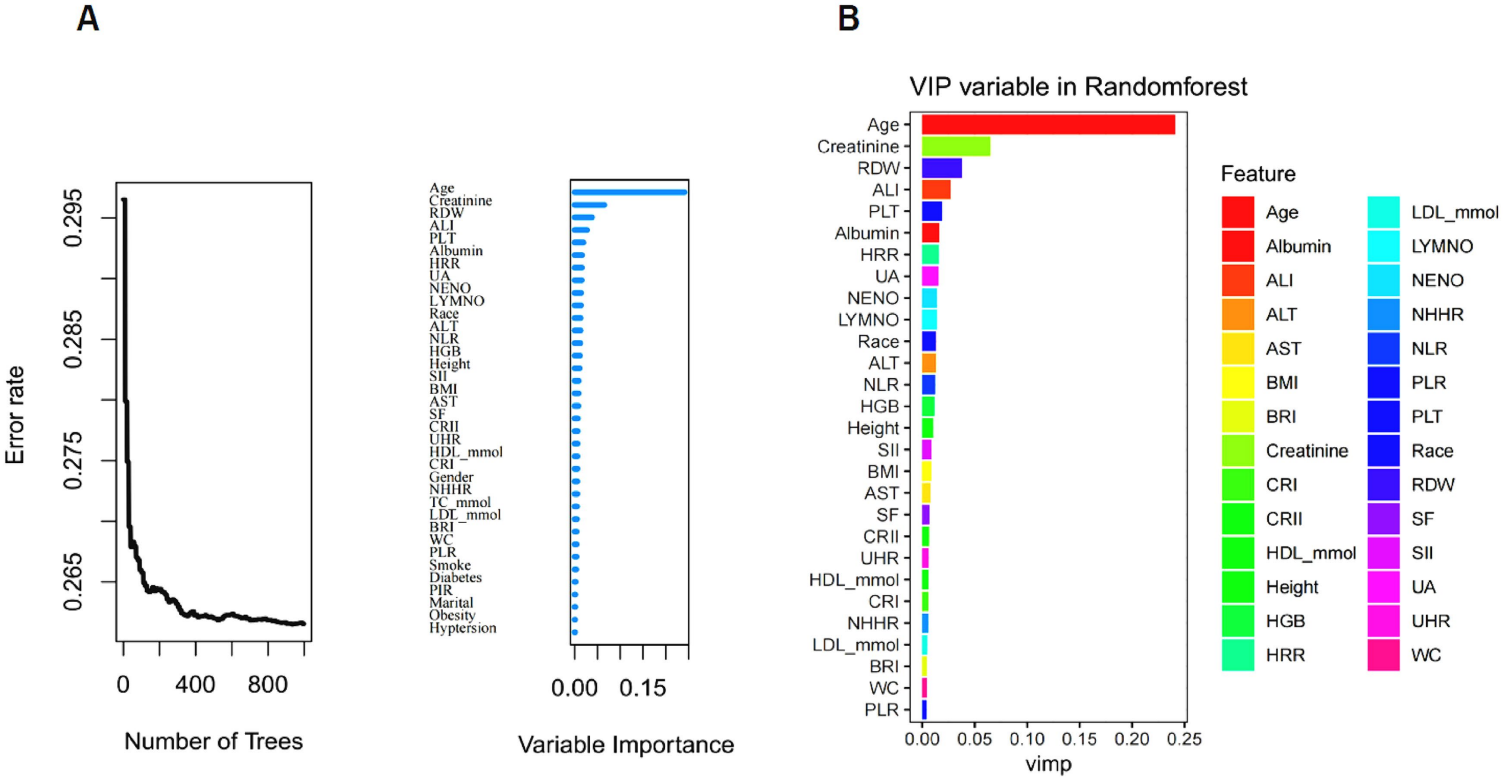
Evaluation of the importance of random forest features. **(A)** The relationship between decision trees and out-of-bag (OOB) error. **(B)** Results of visualizing the importance of characteristic variables in the random forest model.

The RF ensemble algorithm was employed to model survival analysis and assess feature importance. A random seed (seed = 123) was set to ensure result reproducibility, and the survival analysis model was constructed using the randomForestSRC package. The decision trees were visualized (decision trees), showing that when the number of trees reached 400, the OOB error rate plateaued, demonstrating stable predictive performance beyond this threshold (Figure 4A). The var.select function was used to identify the most important features in the random forest model, with age, creatinine, and RDW ranking as the top three. A higher feature importance score suggests a greater impact of that feature on the model’s predictive performance. Finally, a bar plot was created using the ggplot2 package to visualize the feature importance (VIP) in the random forest model (Figure 4B).

### 3.5 Feature variables identified through multiple algorithm-based selection

**Figure 5:**
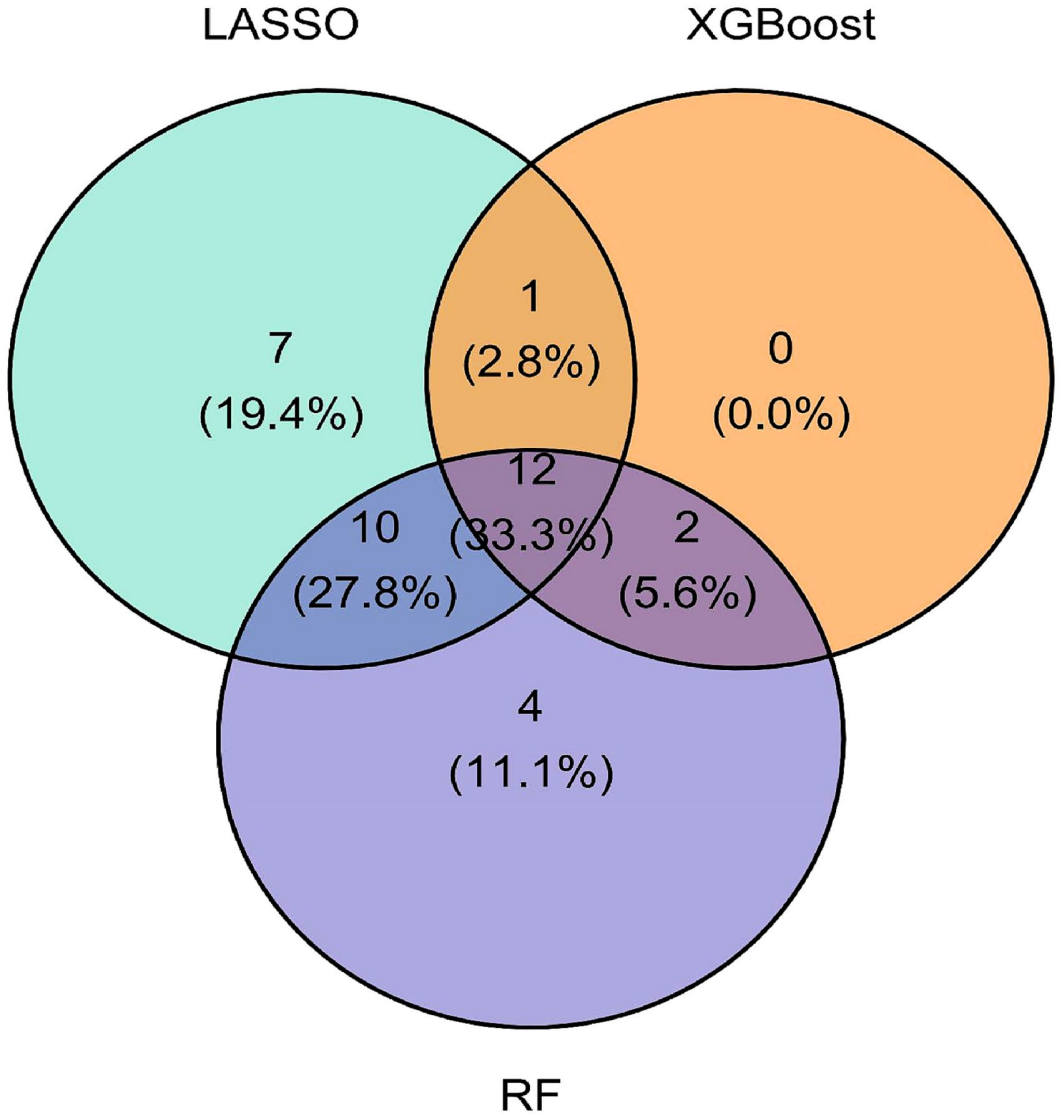
Venn diagram (LASSO regression, XGBoost, random forest).

The feature variables selected by LASSO regression, XGBoost algorithm, and RF were intersected using a Venn diagram, identifying 12 variables, as shown in Figure 5. These include Age, Height, BMI, LDL-c, Albumin, ALT, Creatinine, PLT, UA, HRR, NLR and AST. This multimodal feature selection strategy employs cross-validation across algorithms to effectively reduce single-model selection bias, enhancing the reliability of selected feature variables and playing a crucial role in constructing a prognostic model for SP patients.

### 3.6 Univariate and multivariate cox regression analyses

Survival analysis was conducted on the feature variables identified by the three ML methods. Univariate and multivariate Cox proportional hazards regression models were employed to evaluate the associations between various variables and SP mortality (Table 2), enabling the identification of key prognostic factors for clinical decision support in SP patients. In this study, BMI, ALT, RDW, and PLT were significant in the univariate Cox regression analysis but lost significance in the multivariate analysis, indicating that other variables might have accounted for or adjusted their effects. Therefore, they cannot serve as predictive factors in SP survival analysis. However, the multivariate Cox regression analysis indicated that Age (HR = 1.092, 95% CI: 1.081–1.102, *p* < 0.001), Height (HR = 1.026, 95% CI: 1.018–1.033, *p* < 0.001), and NENO (HR = 1.095, 95% CI: 1.054–1.138, *p* < 0.001) were still significantly associated with an increased risk of mortality in SP patients. HRR (HR = 0.250, 95% CI: 0.130–0.449, *p* < 0.001) was recognized as a protective factor for SP patients. Although UA (HR = 1.001, 95% CI: 1.000–1.002, *p* < 0.008) and Creatinine (HR = 1.001, 95% CI: 1.000–1.002, *p* < 0.004) had a relatively minor impact on the prognosis of SP patients, their increase should not be overlooked as a contributing factor to heightened mortality risk.

**Table 2:** Results of univariate and multivariate cox regression analyses.

Variable	All (Mean ± SD)	HR (univariable)	HR (multivariable)
Age	72.2 ± 8.0	1.10 (1.09–1.11, *p* < 0.001)	1.092(1.081–1.102, *p* < 0.001)
Height	160.1 ± 9.1	1.02 (1.01–1.02, *p* < 0.001)	1.026(1.018–1.033, *p* < 0.001)
BMI	30.2 ± 5.7	0.96 (0.95–0.97, *p* < 0.001)	0.993 (0.981–1.006, *p* = 0.130)
ALT	22.9 ± 12.2	0.98 (0.97–0.99, *p* < 0.001)	1.000 (0.990–1.000, *p* = 0.335)
Creatinine	86.2 ± 53.0	1.00 (1.00–1.00, *p* < 0.001)	1.001 (1.000–1.002, *p* = 0.004)
SF	14.9 ± 5.8	0.99 (0.98–1.00, *p* = 0.040)	1.003 (0.991–1.015, *p* = 0.578)
NENO	4.5 ± 1.6	1.09 (1.05–1.13, *p* < 0.001)	1.095(1.054–1.138, *p* < 0.001)
RDW	13.2 ± 1.5	1.16 (1.13–1.19, *p* < 0.001)	1.013 (0.962–1.066, *p* = 0.745)
PLT	255.4 ± 74.2	1.00 (1.00–1.00, *p* < 0.001)	0.999 (0.998–1.000, *p* = 0.073)
UA	345.4 ± 89.7	1.00 (1.00–1.00, *p* < 0.001)	1.001 (1.000–1.002, *p* = 0.008)
HRR	1.1 ± 0.2	0.16 (0.11–0.23, *p* < 0.001)	0.250 (0.130–0.449, *p* < 0.001)

### 3.7 Visual the forest plot

**Figure 6:**
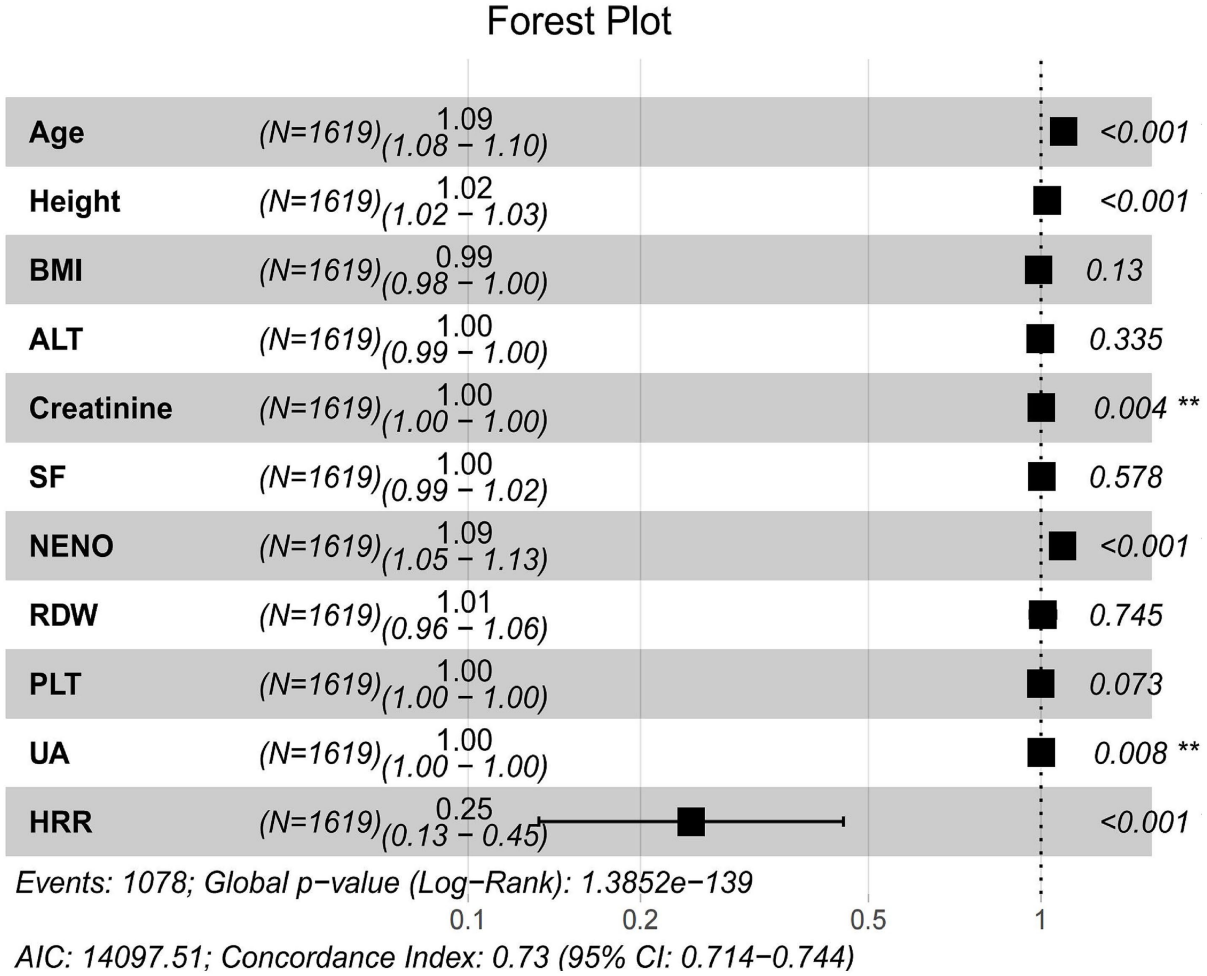
Forest plot visualization.

The forest plot is a widely used visualization tool in survival analysis for illustrating hazard ratios and the statistical significance of variables in Cox regression analysis. The variables selected through univariate and multivariate Cox regression analyses were depicted in a forest plot to visualize their hazard ratios and confidence intervals for mortality risk in older adult SP patients. As illustrated in Figure 6, the forest plot demonstrates a strong association between Age, Height, NENO, HRR, Creatinine, and UA and the risk of mortality in SP patients (p < 0.05). BMI, ALT, SF, RDW, and PLT were not significantly associated with SP-related mortality risk (*p* > 0.05). Additionally, the model demonstrated good overall fit (AIC: 14097.51; Concordance Index: 0.73, 95%CI: 0.714–0.744), indicating its strong predictive capability for SP mortality risk.

### 3.8 Nomogram construction and assessment

**Figure 7:**
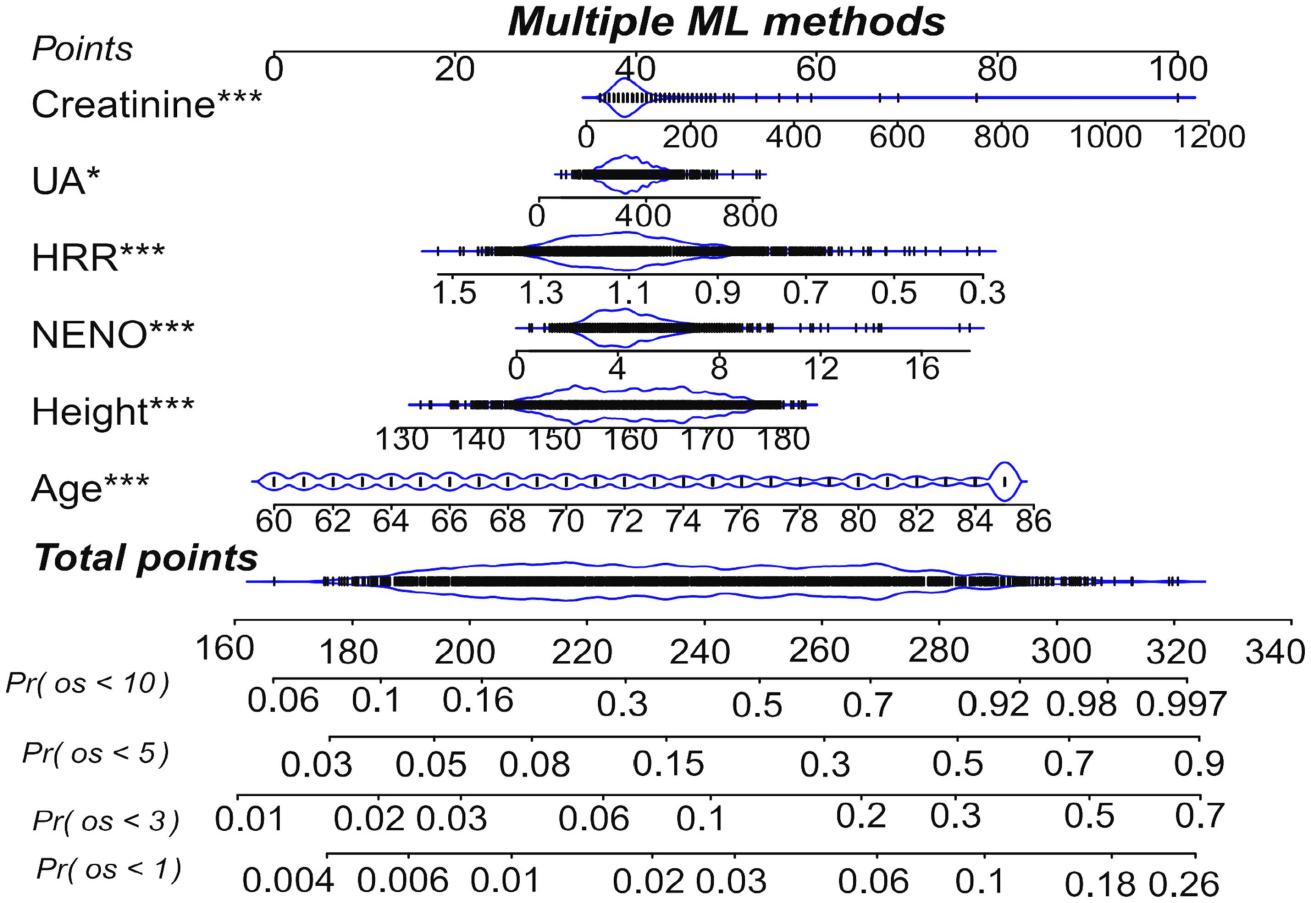
Nomogram Pr(os<1): probability of survival at 1 year; Pr(os<3): probability of survival at 3 years; Pr(os<5): probability of survival at 5 years; Pr(os<10): probability of survival at 10 years.

Based on the multivariate Cox regression model results, the variables Age, Height, NENO, Creatinine, UA, and HRR were incorporated into the nomogram model (Figure 7), with the total score axis summing the scores of each variable into an overall score ranging from 160 to 340. A higher total score signifies an increased risk of adverse events. As shown in the nomogram, an increase in Age, Height, NENO, Creatinine, and UA is associated with a higher score, indicating an elevated mortality risk in SP patients. However, a higher HRR corresponds to a lower score, indicating that HRR is a protective factor in SP patients. This nomogram enables the calculation of a total score based on individual variable values (Age, Height, HRR, NENO) in SP patients, allowing for the identification of high-risk individuals and the prediction of overall survival probabilities at 1, 3, 5 and 10 years, thus guiding personalized treatment strategies.

### 3.9 Plotting the time-dependent ROC curve

**Figure 8:**
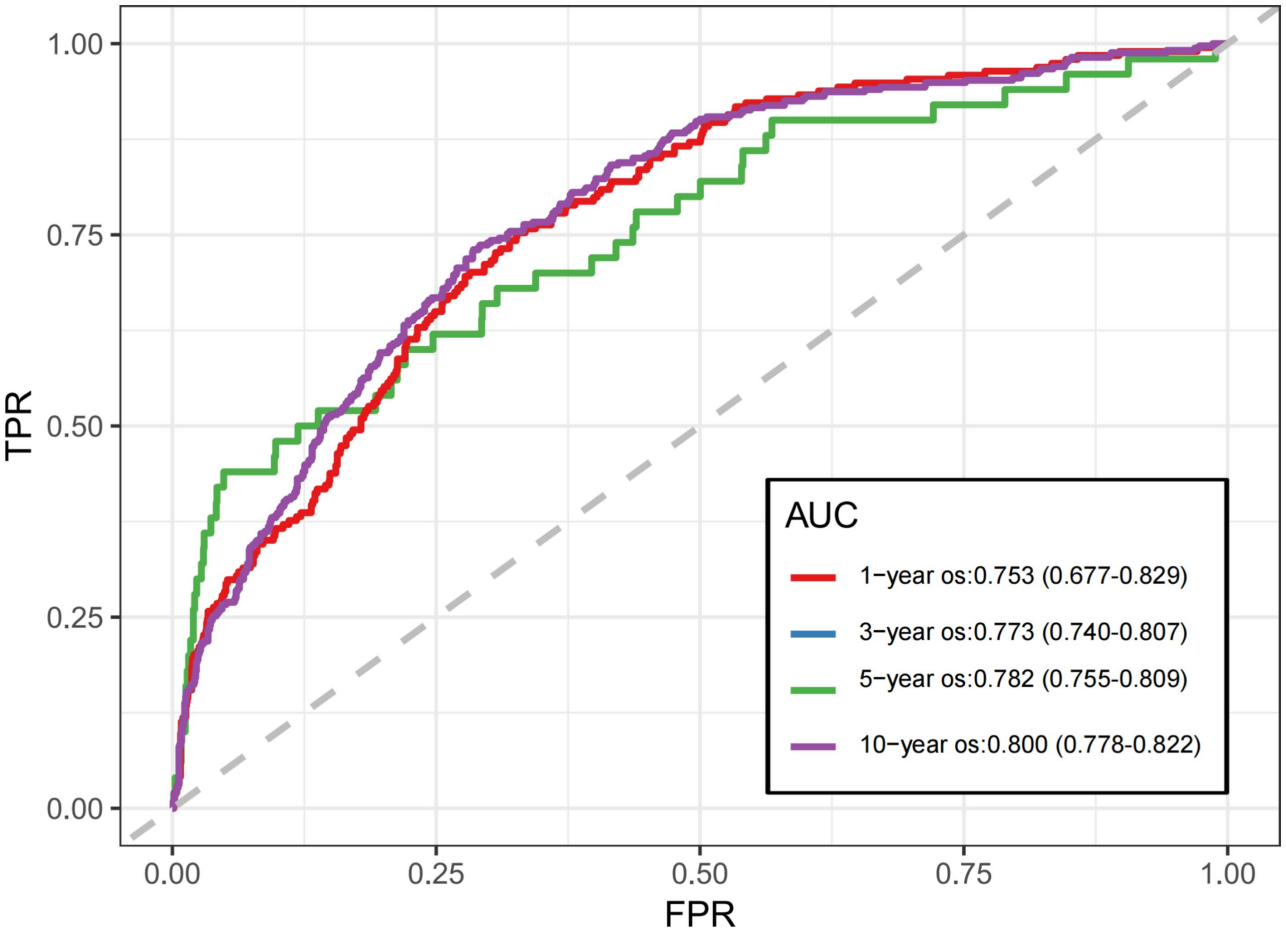
Area under the ROC curve year os: 0.753 (95%CI: 0.677–0.829); 3-year os: 0.773 (95%CI: 0.740–0.807); 5-year os: 0.782 (95%CI: 0.755–0.809); 10-year os: 0.800 (95%CI: 0.778–0.822).

The model’s discrimination was evaluated by computing the nomogram risk score and the time-dependent ROC curve. The time-dependent ROC curve allows a dynamic assessment of the model’s predictive performance across different time points. As illustrated in Figure 8, the time-dependent ROC analysis for SP patients resulted in AUC values of 0.753 (95%CI: 0.677–0.829), 0.773 (95%CI: 0.740–0.807), 0.782 (95%CI: 0.755–0.809), and 0.800 (95%CI: 0.778–0.822) at 1, 3, 5, and 10 years, respectively, demonstrating good discrimination at all evaluated time points. The highest AUC value was observed at the 10-year, suggesting that the model performs best for long-term prediction. Moreover, we implemented DeLong’s test for pairwise comparison of AUCs at 1, 3, 5, 10 year intervals (Table 3). The observed AUC variations across time points were minimal (0.009–0.047), indicating non-significant differences in predictive performance across temporal endpoints. Moreover, AUCs exceeded 0.7 at 1-year, 3-year, and 10-year time points, demonstrating temporal stability in prognostic accuracy. Therefore, nomogram model effectively predicts the mortality risk of SP patients at different time points.

**Table 3:** Results of DeLong.

Comparison groups (year)	95%CI	*p* value
1 vs. 3	−0.044 ~ 0.082	0.647
1 vs. 5	−0.042 ~ 0.099	0.512
1 vs. 10	−0.027 ~ 0.123	0.274
3 vs. 5	−0.018 ~ 0.030	0.727
3 vs. 10	−0.008 ~ 0.060	0.221
5 vs. 10	−0.009 ~ 0.043	0.221

### 3.10 Plotting the calibration curve

**Figure 9:**
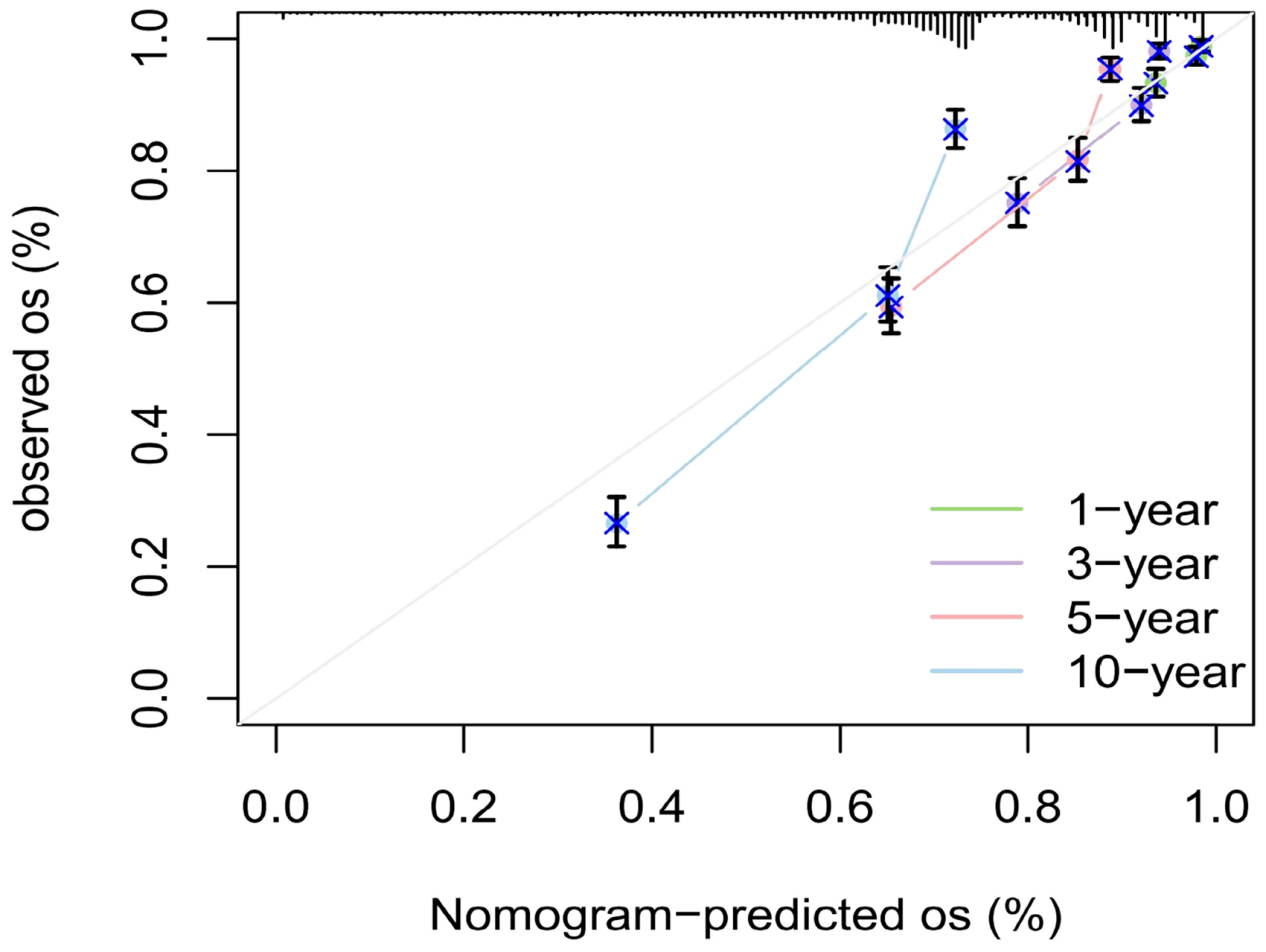
Calibration curves total patients: 1619; Risk patients at time points: 1-year = 1,569, 3-year = 1,429, 5-year = 1,285, 10-year = 947.

To assess the calibration performance of the nomogram model in predicting overall survival (OS) among SP patients. The calibration curve was constructed using the “calibrate” function, and the consistency between predicted and observed values was evaluated using the Bootstrap method (resampling *n* = 1,000). Figure 9 presents the calibration curves for 1, 3, 5 and 10-year survival. For 1-year survival, the predicted probability of 0.90 corresponded to an observed probability of 0.89 (95% CI: 0.87–0.91), resulting in an absolute deviation of −1.0%. For 3-year survival, a predicted value of 0.80 matched an observed value of 0.81 (95% CI: 0.78–0.84), with a deviation of +1.0%. For 5-year survival, the predicted probability of 0.70 corresponded to an observed value of 0.69 (95% CI: 0.65–0.73), with a deviation of −1.0%. For 10-year survival, the predicted value of 0.60 aligned with an observed probability of 0.58 (95% CI: 0.54–0.62), resulting in a deviation of −2.0%. The greatest deviation occurred in the high-prediction range of the 10-year survival curve, where a predicted probability of 0.90 corresponded to a deviation of −3.0%. Nonetheless, all deviations remained within clinically acceptable limits (<5%). This discrepancy may be attributed to the relatively small sample size within the high-survival probability range, which may limit the model’s predictive accuracy in this subgroup. Increasing the sample size in future studies could enhance the model’s stability and reliability. Overall, the nomogram demonstrated excellent calibration performance across the 1- to 10-year follow-up period and provides reliable survival estimates to support clinical decision-making.

### 3.11 Establishing a Kaplan–Meier curve

**Figure 10:**
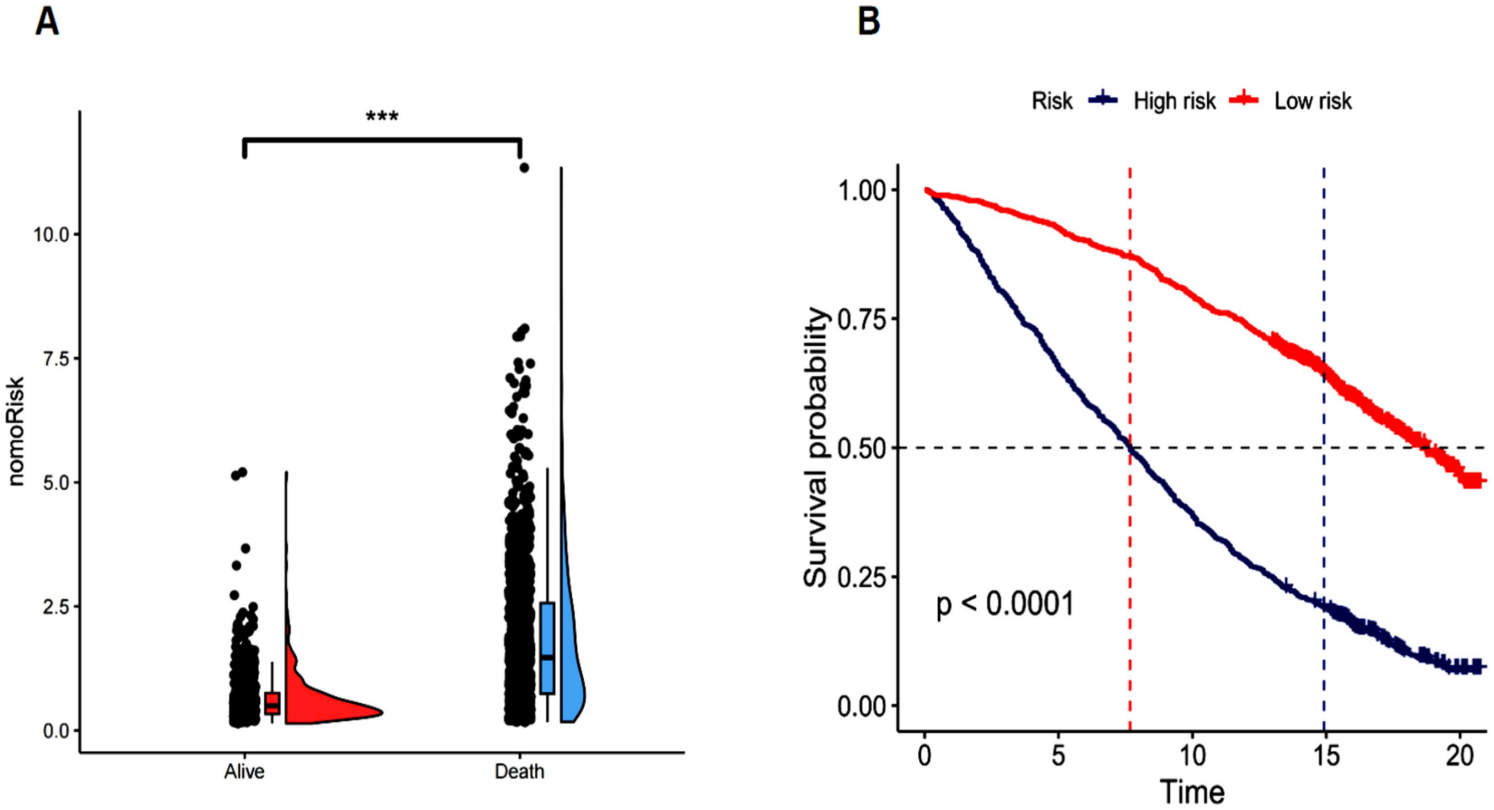
**(A)** Raincloud plot. **(B)** Kaplan-Meier curve.

The nomogram risk score was computed for each patient, and a raincloud plot (Figure 10A) was drawn to visualize the distribution of nomogram risk scores across different outcome groups (Alive and Death). The plot demonstrates that the risk scores in the deceased group are markedly higher than those in the surviving group, suggesting that the nomogram model differentiates between high-risk and low-risk patients. The cohort was stratified into high-risk and low-risk groups based on the median risk score, and survival curves were generated using the “ggsurvplot” function. As shown in Figure 10B, the survival curve of the high-risk group declines more rapidly, with a shorter median survival time, indicating a significantly decreasing survival probability over time. Conversely, the survival curve for the low-risk group exhibits a slower decline and a longer median survival time, indicating a more gradual decrease in survival probability. Moreover, the survival curves for the high-risk and low-risk groups are separated, demonstrating a significant difference in survival probabilities (*p* < 0.0001). Thus, the nomogram model demonstrates efficacy in prognosticating SP outcomes and can be applied in clinical practice to identify high-risk patients, facilitating timely interventions to lower mortality rates.

### 3.12 Establishing decision curve analysis

**Figure 11:**
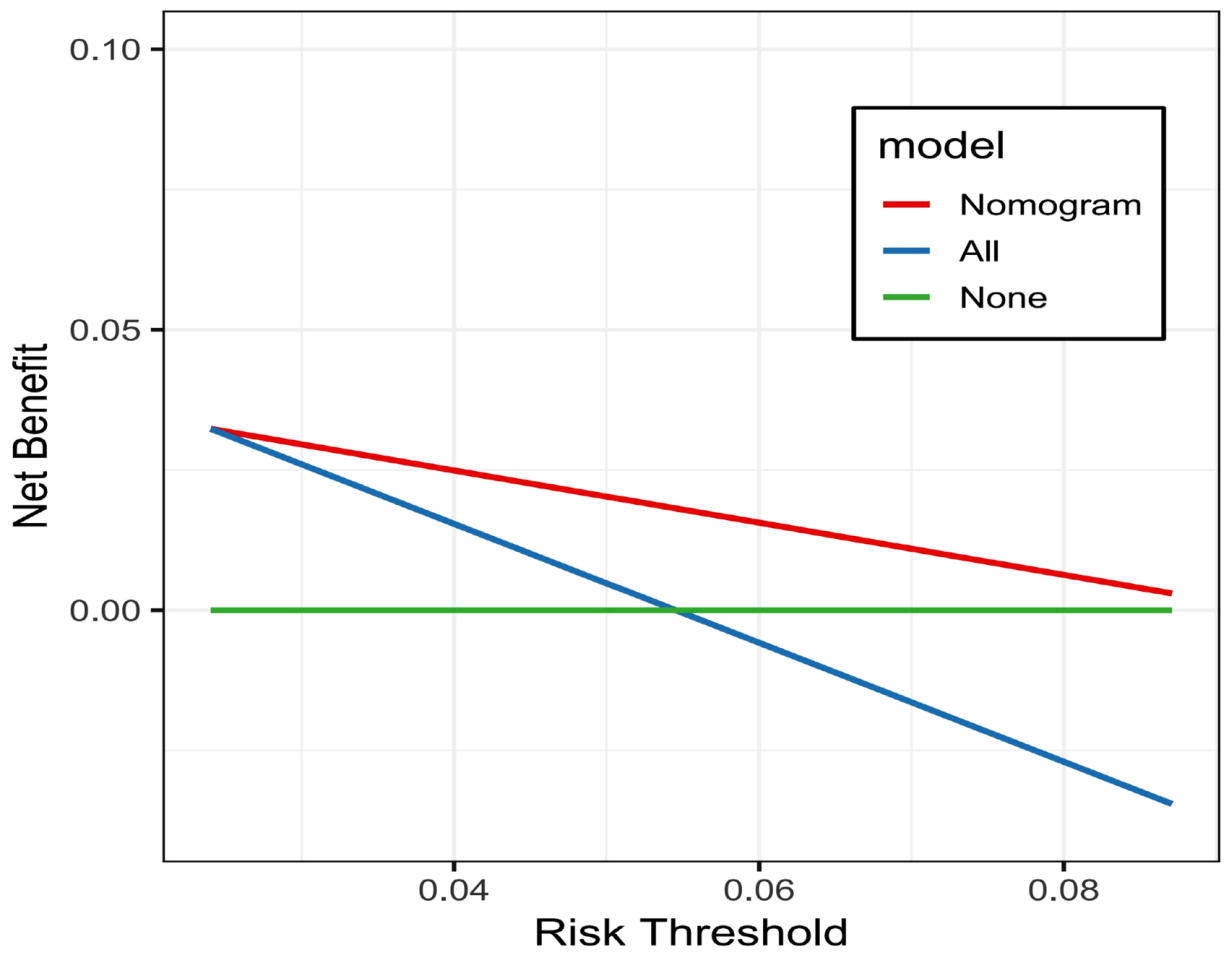
Decision curve analysis all: assumes that interventions are applied to all patients, where net benefit is determined by subtracting the weighted cost of false positives from the true positive rate. None: assumes no intervention for any patient, leading to a constant net benefit of zero.

To evaluate the clinical utility of the nomogram model in predicting survival outcomes in older adult SP patients, we plotted a decision curve analysis (DCA) graph. As shown in Figure 11, within the risk threshold range of 0.02–0.08, the Nomogram and All models performed well, yielding a higher net benefit than None. This result indicates that the Nomogram model has high clinical utility within this threshold range, assisting clinicians in better weighing the benefits and risks of interventions.

## 4 Discussion

This study utilized three distinct ML algorithms—LASSO regression, XGBoost, and RF to identify biomarkers associated with survival prediction in older adult patients with SP. This study is the first to provide a quantitative insight into the association between HRR and SP survival. By incorporating multimodal data—including demographics, anthropometrics, lifestyle factors, and biomarkers—a dynamic nomogram was developed to visualize different variables, enabling clinicians to understand patient conditions better intuitively. This model exhibited strong consistency and accuracy, proving highly effective in differentiating high-risk from low-risk patients. In conclusion, this model aids clinicians in swiftly identifying high-risk older adult patients with SP, allowing for enhanced monitoring and proactive management of modifiable risk factors.

Although SP is prevalent among the older adult, it remains underrecognized in clinical practice. Furthermore, most existing models are designed to predict the risk of SP onset in the older adult, with limited efforts directed toward developing risk stratification and prognostic models for patients already diagnosed with SP. Additionally, these models may fail to comprehensively capture the multifactorial nature of individual patient risk profiles (12). This study incorporates demographic characteristics, lifestyle factors, conventional SP risk indicators, and inflammatory and metabolic biomarkers—such as HRR and CRI—to markedly improve the accuracy of short-term and long-term prognostic predictions in older adult sarcopenic patients (34).

Among the various reported risk factors for SP, increasing age is arguably the most significant. In line with previous studies, our findings indicate that age serves as an independent risk factor for predicting mortality in sarcopenic patients (35). Among the various reported risk factors for SP, increasing age is arguably the most significant. In line with previous studies, our findings indicate that age serves as an independent risk factor for predicting mortality in sarcopenic patients. Moreover, family status, lifestyle habits, physical inactivity, and malnutrition have been strongly linked to SP. (36–38) Notably, multiple evidence-based approaches to managing SP have demonstrated that biomarkers are essential for assessing and managing sarcopenic patients (39).

However, the underlying pathological mechanisms of SP are still not well understood (40). One widely accepted mechanism underlying the onset and progression of SP is age-related chronic low-grade inflammation, known as “inflammaging” (41). With ageing, macrophage activation increases, leading to a chronic subclinical inflammatory state in older adults characterized by elevated pro-inflammatory cytokines and reduced anti-inflammatory cytokines. Inflammation impairs skeletal muscle structure and function in older adults, thereby promoting the onset, progression, and potentially fatal consequences of SP. Research by Jagadish et al. demonstrated that IL-6 and TNF-αare linked to muscle strength and mass reductions (42). *In vitro* studies suggest that IL-6 facilitates muscle atrophy by disrupting anabolic metabolism and energy homeostasis while directly mediating muscle catabolism. Despite substantial research, the association between conventional inflammatory markers (TNF-α, IL-6) and SP remains debated, especially among patients with other age-related diseases (43).

Therefore, identifying more representative biomarkers is an important area of ongoing research. Importantly, this study is the first to explore the potential link between HRR and SP, demonstrating that HRR is a protective factor for sarcopenic patients, where higher HRR values correlate with a lower mortality risk. Moreover, in the nomogram risk scoring system, HRR has a high score and contributes the most to predicting mortality risk in older adult sarcopenic patients. HRR is derived by calculating the ratio of haemoglobin to red cell distribution width. RDW is a straightforward parameter in routine blood tests that indicates variations in circulating red blood cell size (44) and is frequently employed in the differential diagnosis of anaemia. Epidemiological research has suggested that RDW may be a reliable predictor of mortality in cardiovascular disease, cancer, and various chronic conditions (45, 46). Increased RDW reflects an inflammatory state closely linked to various biological processes, including ageing, oxidative stress, nutritional deficiencies, and renal dysfunction (47–49). Junghoon Kim et al. discovered that increased RDW is linked to the onset of SP, especially among overweight and obese individuals (46). Consequently, HRR encapsulates prognostic insights from Hb and RDW, serving as a more reliable prognostic marker than either parameter alone and providing enhanced predictive utility (50).

Likewise, hyperuricemia is strongly linked to systemic inflammation and exhibits oxidative properties. Research indicates that higher uric acid levels elevate the risk of muscle strength decline (51). A study conducted on Japanese postmenopausal women revealed that hemodialysis patients with reduced muscle mass exhibited lower serum creatinine levels, suggesting a positive association between serum creatinine and the onset and progression of SP. Hence, in alignment with prior research, our study confirms that uric acid and creatinine serve as independent risk factors for mortality in sarcopenic patients.

However, a single predictive factor is insufficient to encapsulate the complexity of SP prognosis and progression. Consequently, this study introduces a dynamic nomogram model by integrating multimodal data, allowing clinicians to estimate the probability of future disease progression based on a patient’s current condition, thus offering a robust and intuitive scientific tool for personalized precision medicine (52–54). By identifying sarcopenic patients at higher risk of mortality, clinicians can utilize this predictive model to optimize patient management and enhance overall care for older adult SP patients. More frequent follow-ups, tailored pharmacological treatments, and targeted lifestyle interventions may be necessary rather than applying uniform treatment and intervention strategies to all sarcopenic patients.

### 4.1 Limitations

We recognize several limitations in this study. First, it may not establish definitive causal relationships as an observational study. Secondly, our model was developed based on data from a single database, which may introduce inherent biases in data collection. Lastly, potential biases may exist as the data utilized in this study were sourced from a U.S. SP cohort. Therefore, further validation across different populations is needed, and refinements may be required to improve predictive accuracy. In the future, we aim to include larger-scale, multicenter SP cohorts to validate the model’s generalizability further.

## 5 Conclusion

In summary, this study established a dynamic visual risk stratification model for predicting the 1-year, 3-year, 5-year, and 10-year mortality risk in older adult SP patients, offering precise prognostic insights. Incorporating multimodal data, the machine learning-based prognostic model exhibits robust predictive accuracy and significant clinical benefits. This enables tailored medical strategies and targeted interventions for high-risk SP patients, ultimately enhancing strategies for SP prevention and healthcare improvement.

## Data Availability

The original contributions presented in the study are included in the article/Supplementary material, further inquiries can be directed to the corresponding author.

## Footnotes

^1^ https://www.cdc.gov/nchs/nhanes/
