# Supplementary material for: Multimodal data-driven prognostic model for predicting long-term outcomes in older adult patients with sarcopenia: a retrospective cohort study
Source: Front Public Health. 2025 Aug 7;13:1614374. doi: 10.3389/fpubh.2025.1614374 (PMC12369588; doi:10.3389/fpubh.2025.1614374)

Supplementary material

1. Decision tress

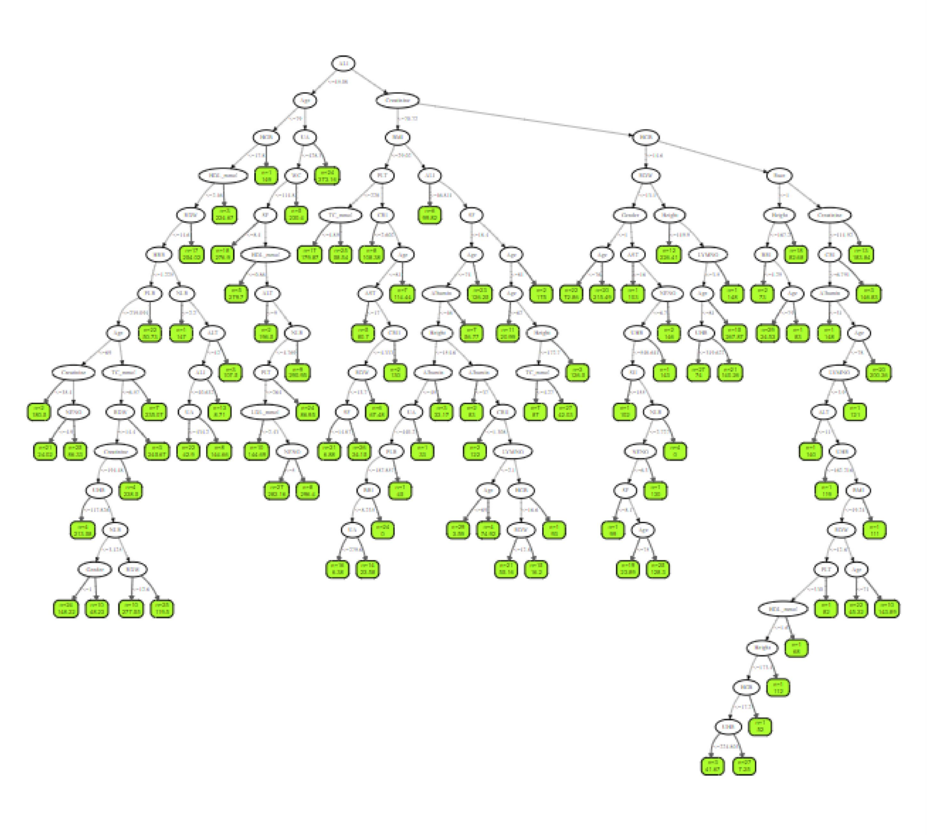

Figure 1a. Box plot of Alive group and the Dead groups

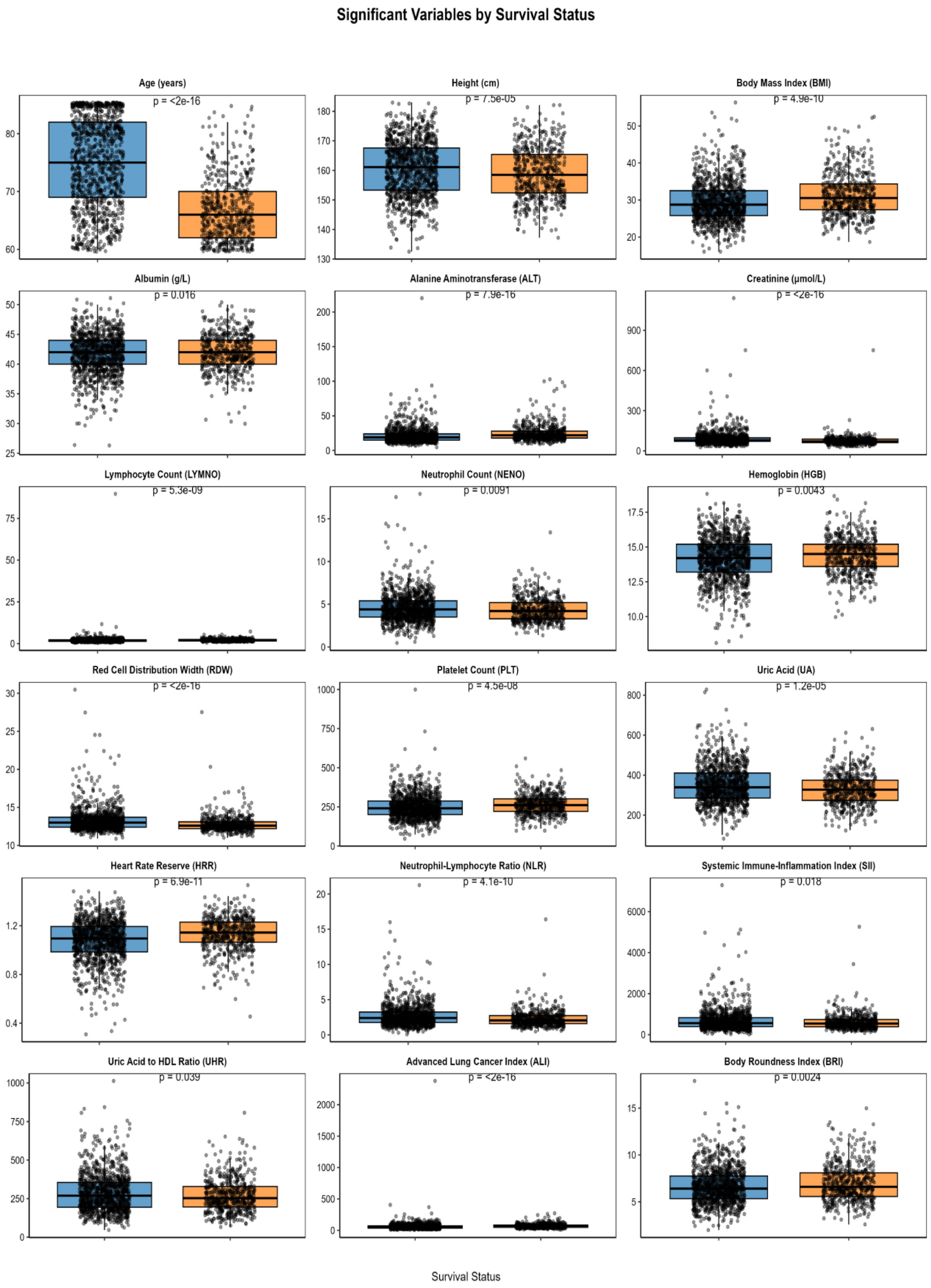

***represents the Alive group, yellow represents the Dead group.**

Figure 1b. Sex-Stratified Box Plots for Important Variables:

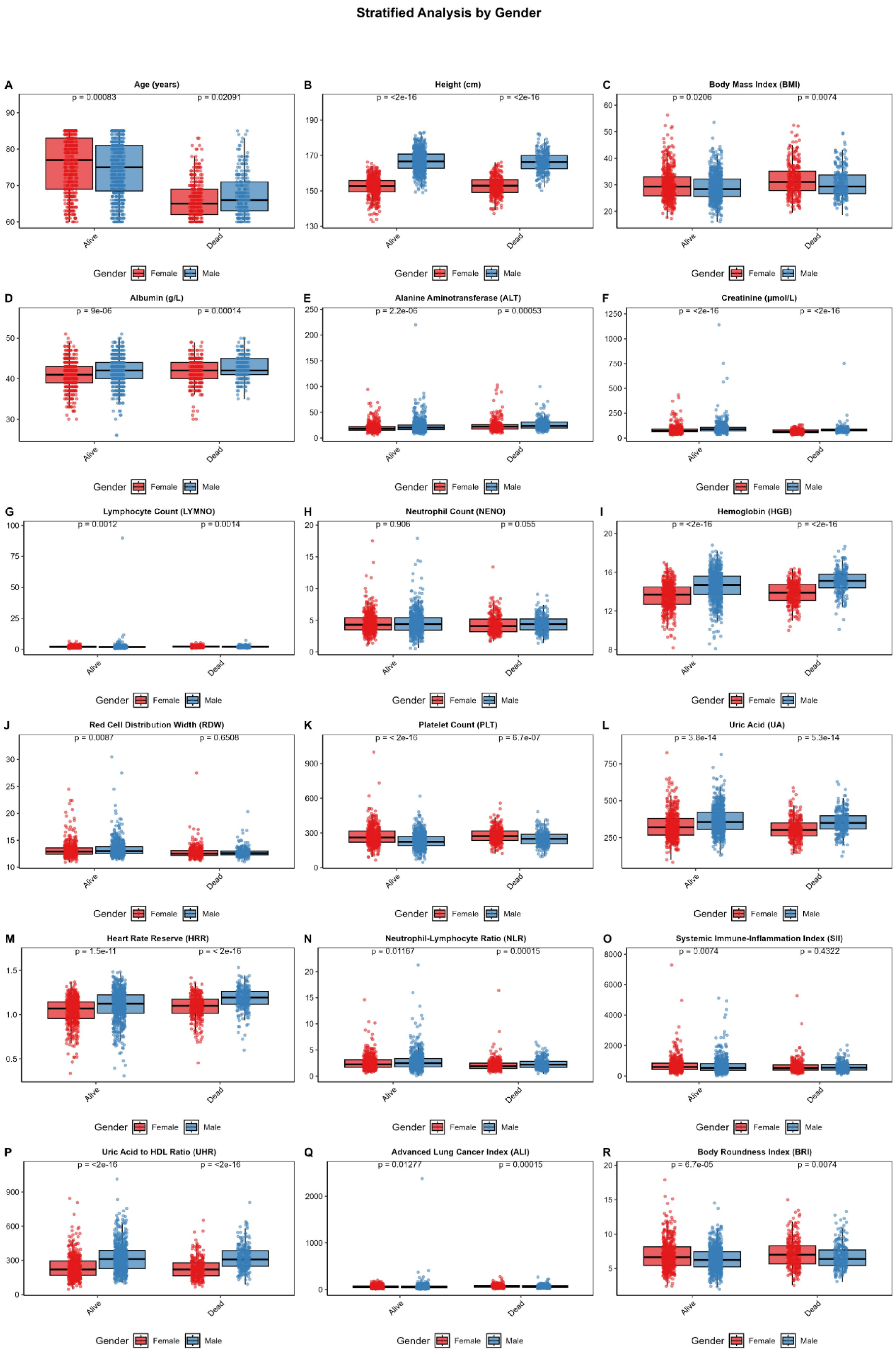

Supplement: Supplementary file 1 [file Supplementary_file_1.pdf]
